# Management of Wilms' tumor: NWTS vs SIOP

**DOI:** 10.4103/0971-9261.54811

**Published:** 2009

**Authors:** Sushmita Bhatnagar

**Affiliations:** Department of Pediatric Surgery, Bai Jerbai Wadia Hospital for Children, Acharya Donde Marg, Parel, Mumbai, India

**Keywords:** National Wilms' tumor study group, Societe internationale D'oncologie pediatrique, Wilms' tumor

## Abstract

With the availability of several protocols in the management of Wilms' tumor, there is dilemma in the minds of the treating oncologists or pediatric onco-surgeons as to whether the child should receive upfront chemotherapy or should be operated upon primarily. It is necessary for us to understand why do we follow either of the protocols, NWTS which follows the upfront surgery principle or the SIOP which follows the upfront chemotherapy principle in all stages of the disease. While deciding which protocol to follow, it is imperative to know the pros and cons of the treatment strategies and also to study the outcome patterns in both the treatment regimes which is what this article highlights. In an attempt to compare all the differences in both the major protocols, it was realized that most of our patients in the Indian scenario present with advanced disease and thus poorer outcomes if intensive and appropriate treatment strategies are not utilized. Hence, it is imperative that we should study our own patients through the Indian Wilms' tumor study group and adopt the policies which improve the overall event free survival on a nationwide basis.

## INTRODUCTION

Wilms' tumor is the most common malignant tumors of the kidney in children. The treatment of Wilms' tumor can be considered as the paradigm for multimodal treatment of malignant solid tumors in childhood. Progress has occurred from the times when this tumor was universally fatal to this era when more than 85% of the patients can be completely cured with localized disease and over 70% for metastatic disease.[1] Major research and randomized controlled trials performed by several co-operative groups have made the future of Wilms' tumor patients very bright. The two major groups which have tremendous contributions in the management of Wilms' tumor are National Wilms' Tumor Study (NWTS) and the Societe Internationale D'oncologie Pediatrique (SIOP). Two other groups that deserve mention are United Kingdom Children's' Cancer Study Group (UKCCSG) and the Children's Oncology Group (COG), which will be briefly discussed herein.

## HISTORICAL REVIEW

Treatment similar to the nomenclature of Wilms' Tumor (nephroblastoma) had a progressive history.[1] Surgical excision, which is the primary method of treatment, initially carried a high operative mortality rate. Simon in Heidelberg in 1871 performed the first planned nephrectomy in an adult patient after establishing the feasibility of unilateral nephrectomy in experiments in dogs.[2] In 1877, Jessop in Leeds, England, performed the first nephrectomy for a Wilms' tumor in a 2-year-old child. William Ladd, the father of American Pediatric surgery, standardized the surgical therapy by refining operative techniques with a resultant decrease in surgical mortality.

In 1950, radiation therapy was added by Friedlander of Cincinnati.[3] As an adjunct to surgical excision and irradiation therapy, intravenous administration of actinomycin-D in 1954 and vincristine in 1963 completed the therapeutic regime.

By combining improved surgical techniques, irradiation therapy and chemotherapy, Farber and his group were able to report a 2-year survival rate of 81%.[4]

## NATIONAL WILMS' TUMOR STUDY GROUP

Subsequent to 1966,[1] amongst the number of published reports, there was insufficient large series data collection on patients with this tumor.

Three co-operative groups, the children's cancer study group (CCSG), the cancer and leukemia group B (CALGB), and the southwest Oncology Group (SWOG) combined to from an intergroup known as National Wilms Tumor Study (NWTS) in 1969 as there was a need to collaborate in gathering a statistically significant number of patients. The NWTS, a cancer research co-operative group, was created with the purpose of improving survival of children with Wilms' tumor. Many pediatric oncology centers (over 250) in the United States, Canada and other countries joined this study group, and a large number of patients were enrolled which accounted for approximately 70%-80% of patients with Wilms' tumor in those countries.

NWTS ran five clinical trials, NWTS-1 to NWTS-5. The first four were randomized trials, whereas NWTS-5 which completed in 2003 was a clinical trial designed to look primarily at biologic prognostic factors and was not randomized.

### Purpose of study

NWTS 1 – To determine the effect of surgical technique on the results of the treatment.

NWTS 2 – To study the prognosis of patients with Wilms' tumor.

NWTS 3 – To reduce the treatment for low-risk patients and find better chemotherapy for those at high risk for relapse.

NWTS 4 – To evaluate the efficacy, toxicity and cost of administration of different regimens for the treatment of Wilms' tumor.

NWTS 5 – To identify the biologic prognostic factors.

In 2001, NWTS merged with several other pediatric oncology cooperative groups to create the Children's Oncology Group (COG). However, the NWTS is still active in name today completing follow-up of the late effects of treatment for patients previously enrolled in its trials (LATE).

## SOCIETE INTERNATIONALE D'ONCOLOGIE PEDIATRIQUE

SIOP (Societe Internationale D'oncologie Pediatrique) is another European Group that in 1971 started studies on Wilms' tumor. It differed from NWTS in the concept of giving preoperative chemotherapy to all patients.

The promoters of SIOP with a view of reducing the risk of tumor rupture during upfront surgery, as was seen during NWTS studies, planned upfront therapy – initially radiotherapy and later chemotherapy to shrink the tumor.

To decrease the need for postoperative abdominal radiation therapy to treat children who have tumor rupture during nephrectomy, SIOP conducted trials in which children received prenephrectomychemotherapy or abdominal radiation therapy. Prenephrectomy abdominal irradiation decreased the percentage of nephrectomies complicated by tumor rupture from 33% (20 of 60 nephrectomies) to 4% (3 of 72 nephrectomies).[5] In a subsequent randomized trial (SIOP-5), the frequency of tumor rupture was nearly the same for children treated with prenephrectomy abdominal irradiation and dactinomycin (8%; 7 of 76 children) and for those treated with prenephrectomy chemotherapy with vincristine and dactinomycin (6%; 5 of 88 children).

The details of several trials conducted by SIOP, including the dates and their conclusions, are as follows:

### SIOP 1 – (09/71)

Pre-operative radiotherapy significantly prevents tumor rupture and induces favorable stage distribution.

Additional actinomycin-D (6x) does not improve DFS/AS in either arm.

### SIOP 2 – (10/74)

The benefits of pre-operative radiotherapy as in SIOP 1 trial were confirmed.

Post-operative chemotherapy for 6 months as good as 15 months. Hence, children should receive chemotherapy only for 6 months following nephrectomy.

### SIOP 5 – (01/77)

Pre-operative 2 drug chemotherapy is as effective as pre-operative radiotherapy in avoiding ruptures and improving the stage distribution.

### SIOP 6 – (07/80)

Stage I – Treatment with vincristine and dactinomycin was as effective for 17 weeks as for 38 weeks in terms of event-free and overall survival rates.

Stage II – Patients with negative lymph nodes who were assigned to receive no radiation therapy had a higher recurrence rate.

### SIOP 9 – (12/87)

Stages I, II, III – 8 weeks pre-operative treatment does not produce a favorable stage distribution compared to 4 weeks.

Stage II – For patients with negative lymph nodes, the rate of relapse was reduced by treatment with epirubicin without radiation therapy.

### SIOP 93-01 – (12/93)

Reduction of postoperative chemotherapy (for intermediate-risk and anaplastic Wilms' tumor) to four doses of vincristine and one dose of dactinomycin was not less effective than standard postoperative chemotherapy according to histologic features.

Two other groups that have done significant studies in the management of Wilms' Tumor are UKCCSG and COG. The UKCCSG began in 1977 when a small number of doctors who had been treating children with cancer joined forces with the aim of improving the management and treatment of children with cancer and advancing knowledge about children's cancers. The UKCCSG recently completed a randomized comparison of these two approaches in its UKW3 trial,[6] which showed a more favorable tumor stage distribution and significant reduction in the overall burden of therapy together with reduced surgical complications after the elective use of prenephrectomy chemotherapy. For these reasons, the UKCCSG has now joined the current SIOP Wilms' tumor 2001 clinical trial and study.[7]

As compared to UKCCSG which started with a small group of doctors, the Children's Oncology Group (COG) started as a worldwide clinical trial cooperative study group for Wilms' as well as other tumors. It was formed in 2000 with the merging of four independent cooperative groups; the Children's Cancer Study Group (CCG), Pediatric Oncology Group (POG), Intergroup Rhabdomyosarcoma Study Group (IRS), and the National Wilms' Tumor Study Group (NWTSG).

Thus, in a nutshell, the NWTSG has always recommended upfront nephrectomy to define the accurate stage of the tumor and the histology, on which further treatment stratification is decided. In contrast, the SIOP investigators pioneered the concept of prenephrectomy chemotherapy in all patients over 6 months of age to reduce the tumor size and prevent intraoperative spillage due to tumor rupture and increased the proportion of children with a lower tumor stage that required less overall treatment.[8]

## STAGING OF WILMS' TUMOR – NWTSG VS SIOP

Staging criteria for Wilms' tumor are based exclusively on the anatomic extent of the tumor, without the consideration of genetic, biologic, or molecular markers.[9,10] Two major staging systems are currently used: An up-front, surgery-based system developed by the NWTSG and an upfront chemotherapy-based system developed by the International Society of Pediatric Oncology (SIOP)[10,11] [Table 1].

**Table 1:** Staging systems for Wilms' tumors

NWTSG (before chemotherapy)	SIOP (after chemotherapy)
Stage I	
a. Tumor is limited to the kidney and completely excised	a. Tumor is limited to kidney or surrounded with fibrous pseudocapsule. If outside the normal contours of the kidney, the renal capsule or pseudocapsule may be infiltrated with the tumor, but it does not reach the outer surface, and is completely resected (resection margins “clear”)
b. Tumor was not ruptured before or during removal	b. The tumor may be protruding into the pelvic system and “dipping” into the ureter (but it is not infiltrating their walls)
c. Vessels of the renal sinus are not involved beyond 2 mm	c. The vessels of the renal sinus are not involved
d. There is no residual tumor apparent beyond the margins of excision	d. Intrarenal vessel involvement may be present
Stage II	
a. Tumor extends beyond the kidney but is completely excised	a. The tumor extends beyond kidney or penetrates through the renal capsule and/or fibrous pseudocapsule into perirenal fat but is completely resected (resection margins “clear”)
b. No residual tumor is apparent at or beyond the margins of excision	b. The tumor infiltrates the renal sinus and/or invades blood and lymphatic vessels outside the renal parenchyma but is completely resected
c. Tumor thrombus in vessels outside the kidney is stage II if the thrombus is removed en bloc with the tumor	c. The tumor infiltrates adjacent organs or vena cava but is completely resected
Although tumor biopsy or local spillage confined to the flank was considered stage II by NWTSG in the past, such events will be considered stage III in upcoming COG studies.	
Residual tumor confined to the abdomen:	
Stage III	
a. Lymph nodes in the renal hilum, the peri-aortic chains, or beyond are found to contain tumor	a. Incomplete excision of the tumor, which extends beyond resection margins (gross or microscopical tumor remains postoperatively)
b. Diffuse peritoneal contamination by the tumor	b. Any abdominal lymph nodes are involved
c. Implants are found on the peritoneal surfaces	c. Tumor rupture before or intra-operatively (irrespective of other criteria for staging)
d. Tumor extends beyond the surgical margins either microscopically or grossly	d. The tumor has penetrated through the peritoneal surface
e. Tumor is not completely resectable because of local infiltration into vital structures	e. Tumor thrombi present at resection margins of vessels or ureter, transected or removed piecemeal by surgeon
	f. The tumor has been surgically biopsied (wedge biopsy) prior to preoperative chemotherapy or surgery
	Regional lymph node involvement was considered stage II in the previous SIOP staging system.
Stage IV	
Presence of hematogenous metastases or metastases to distant lymph nodes	Hematogenous metastases (lung, liver, bone, brain, etc.) or lymph node metastases outside the abdominopelvic region
Bilateral renal involvement at the time of initial diagnosis	Bilateral renal tumors at diagnosis

COG-Children's Oncology Group; NWTSG-National Wilms' Tumor Study Group; SIOP-International Society of Pediatric Oncology

Both staging systems have proven valuable in predicting outcomes; however, because of the difference in the timing of nephrectomy, a stage-wise comparison becomes a limitation.

## HISTOLOGIC DIFFERENCES

Histologic classification defined by the NWTSG and SIOP studies differs because the SIOP protocols use preoperative chemotherapy. Anaplasia is the basis of histologic classification in NWTS, whereas the SIOP histological classification is based on cell differentiation, including chemotherapy induced “regressive” changes. However, it has been proved that anaplastic features are not altered even after chemotherapy. Hence, the histologic classification of SIOP provides adequate risk stratification. The three groups proposed by the revised SIOP histologic classification are as follows:

- Low risk (completely necrotic nephroblastoma or cystic partially differentiated nephroblastoma),
- Intermediate risk (regressive, epithelial, stromal, mixed, or focal anaplastic nephroblastoma), and
- High risk (blastemal or diffuse anaplastic nephroblastoma).[11,12]

## TREATMENT OPTIONS – NWTSG VS SIOP

Surgical excision of the tumor, combination chemotherapy and radiotherapy, all play an important part in the treatment of Wilms' Tumor. The essential difference between the NWTS and SIOP treatment protocols is that the latter routinely gives preoperative chemotherapy.

The recommended algorithm for management of WT as per NWTSG is Clinical staging → Surgical Staging (Histological Diagnosis); details are given in Table 2.

**Table 2:** Management of Wilms' tumor as per NWTS protocol

Stage	Treatment
Stage I FH/UH	18 weeks of DAM/VCR
Stage II FH	18 weeks of DAM/VCR
Stage III + IV FH	24 weeks of DAM/VCR/DOX, RT tumor bed + involved sites
Stage II–IV UH	24 weeks of DAM/VCR/DOX/CPM/Etoposide, RT tumor bed + involved sites

DAM-Dactinomycin; VCR-Vincristine; DOX-Doxorubicin; CPM-Cyclophosphamide; RT-Radiotherapy

The algorithm for management of Wilms' tumor as per SIOP 93-01 protocol is as shown in Table 3. The post-operative therapy involves chemotherapeutic drugs that vary as per the stage of the tumor with or without involvement of lymph nodes and the use of radiotherapy as per the stage and grade of the tumor; the details of are shown in Table 4.

**Table 3:** Management of Wilms' tumor as per SIOP protocol

Clinical staging		
Localized	4 weeks of DAM/VCR.	Surgical staging
Metastatic	6 weeks of DAM/VCR/EPI	(Histological diagnosis)

DAM-Dactinomycin; VCR-Vincristine; EPI-Epirubicin

**Table 4:** Regime of post-operative therapy as per SIOP protocol

	Stage	Treatment
Localized	Stage I, Low grade	none
	Stage I, Intermediate grade + anaplasia	18 weeks DAM/VCR
	Stage II– (no lymph nodes)	28 weeks DAM/VCR/EPI
	Stage II + and III	28 weeks DAM/VCR/EPI + RT tumor bed.
	High grade	34 weeks EPI/IF/VP16/CARBO + RT
Metastatic	IV	As per the local stage for tumor + treatment of metastases – RT and/or excision.

DAM- Dactinomycin; VCR-Vincristine; EPI-Epirubicin; IF-Ifosfamide; VP-16, Etoposide; Carbo-Carboplatin; RT-Radiotherapy

## PROS AND CONS

NWTSG investigators recommend immediate nephrectomy because prenephrectomy chemotherapy administration is associated with several risks, including the following:

1. administration of chemotherapy to a patient with a benign disease as in SIOP trials, pre-chemotherapy confirmation of diagnosis is not mandatory;
2. administration of chemotherapy to a patient with a different histological type of malignant tumor;
3. modification of tumor histology; and
4. loss of staging information.

**Figure 1:**
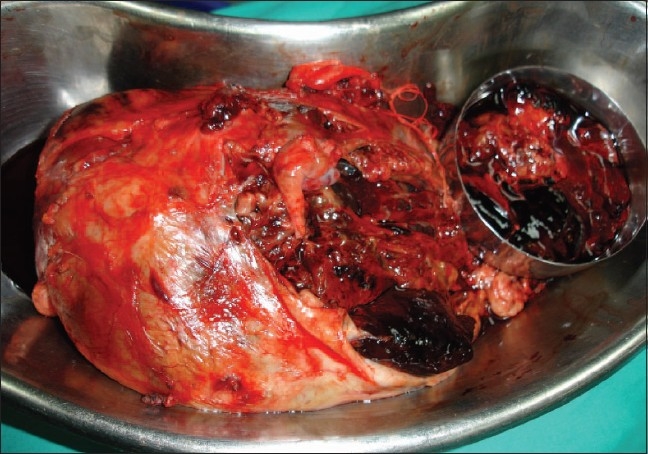
Intra-operative tumor spillage in a child treated as per NWTS protocol

On the other hand, apart from the risk of surgical complications, the NWTSG has the following disadvantages:

1. Tumor spillage intra-operatively, which increases the risk of local abdominal relapse and subsequent poor outcome[13] [Figure 1].
2. Failure to sample lymph nodes leads to downstaging and under-treatment of the patient.[13]

SIOP investigators are of the opinion that prenephrectomy chemotherapy has a major advantage in that

**Figure 2:**
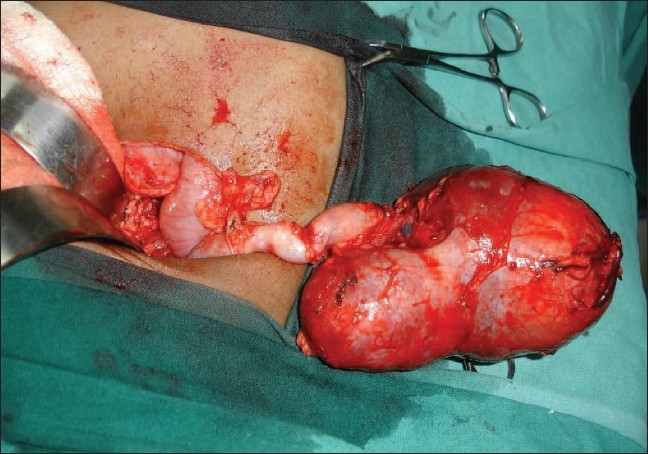
Marked reduction in size of the tumor following chemotherapy as per SIOP protocol

1. It reduces the tumor size considerably, thereby making surgery simpler and reducing the chances of tumor rupture intra-operatively; this reduces the likelihood of local and distant recurrence [Figure 2].
2. Possible role of renal sparing surgery in the affected kidney could be evaluated with the tumor size reduced pre-operatively.[14]

Pre-nephrectomy chemotherapy is considered by some to cause alterations in tumor histology and to downstage the tumor. The arguments of the SIOP against the problems identified with pre-nephrectomy chemotherapy include the following:

1. New risk groups for the purpose of treatment stratification can be identified through modification in tumor histology that occurs post-chemotherapy.[11,15]
2. Since the overall burden of tumor (loss of staging) is reduced with pre-operative chemotherapy, the use of anthracyclines and radiotherapy can be restricted to children whose tumors do not adequately respond to the relatively simple first-line chemotherapy.

Often the treatment regimens for Wilms' tumor have been compared; however, since the staging systems are not equivalent, the regimens are bound to be different. Albeit, it is important to know the differences in the regimens so as to be able to choose the protocol for the institute.[12,15–17]

## OUTCOME – NWTSG VS SIOP

The largest numbers of patients have been studied in the several trials of both NWTS and SIOP groups though the UK group has also been active in the trials of Wilms' tumor. Hence, this review focuses on their findings. Both treatment approaches yield almost equivalent clinical outcomes though a valid debate continues about the relative merits of each approach.[18]

## BILATERAL WILMS' TUMOR

Bilateral WT can be synchronous or metachronous. Synchronous Wilms' account for about 6%-7% of the tumors in the Western literature.[19–21] Prior to the NWTS and the SIOP trials, the management of bilateral Wilms' tumor involved radical surgical procedures, which lead to patients on dialysis and subsequent renal transplants and poor outcome.[19] SIOP has been recommending pre-operative chemotherapy without biopsy for all renal tumors including bilateral Wilms' tumor.[22] In NWTS-4 trials, pre-operative chemotherapy was studied for bilateral Wilms' tumor with a view to salvage the kidneys as much as possible.[23] Thus, in recent reports, a very conservative surgical approach has been stressed.[23,24] After the trials, the NWTS recommends bilateral biopsies followed by pre-operative chemotherapy and then renal salvage surgery.[25] For the renal salvage surgery to be successful, at least two-third of one kidney must remain post-operatively. Several surgical options exist, and each patient needs to be individualized as to the choice of surgical procedure depending on the affection of the kidneys in terms of tumor volume. Bilateral nephrectomies followed by renal transplant becomes mandatory when bilateral partial nephrectomies, unilateral radical nephrectomy with opposite partial nephrectomy as renal salvage procedures, or in cases where the tumor does not respond to chemotherapy and salvage of the kidney is not possible.

## LAPAROSCOPY IN WILMS' TUMOR

Minimally invasive surgery in the pediatric patients with cancers is gaining popularity. It is being utilized for laparoscopic biopsies, diagnosis of the tumor extent, second-look, as well as for the complete excision of the tumors. Though the role of laparoscopy in the present times is limited in tumor resection surgery, more and more reports are available in favor of laparoscopic tumor resection.[25–27] Though the studies are not done over a long term and for a large group of patients, the preliminary reports are not indicative of port-site recurrence of malignancy.[28]

## METASTATIC WILMS' TUMOR

Hematogenous metastases of WT occurs in lung, liver, bone, brain, or lymph nodes outside the abdomen, of which lungs are the most common site of metastases. NWTS and SIOP recommend different protocols in the management of lung metastases.[6] NWTSG utilizes both CT scan and X-ray chest to confirm the presence of lung metastasis. The CT-only nodules do not mandate treatment with whole-lung irradiation as per the NWTS 5 study conclusions. The 2-year relapse-free survival estimate for patients with stage IV disease treated on NWTS 4 was 81%.[29] In contrast, X-ray chest was considered diagnostic for lung metastases by SIOP studies and either chemotherapy or surgical resection of metastases was undertaken. With this approach, SIOP reported a 4-year event free survival rate of 83%.[30] The UK group UKCCSG though did not have results as good as SIOP when radiation therapy was not given for lung metastases, and reported a survival rate of only 65%.[30] SIOP has reserved lung irradiation for those who are non-responders to other forms of therapy. Assessment of the need of lung irradiation is yet to be studied and will be done in the future COG trials.

## MANAGEMENT OF CHILDREN WITH AGE LESS THAN 6 MONTHS

For children with age less than 6 months, SIOP has a different protocol as these children cannot always be given upfront chemotherapy. Also, as their prognosis is better, a less aggressive treatment is required. The treatment suggested is as follows:

1. For the majority, surgery first is indicated for the following reasons:
  - Small- or medium-sized tumors easily removable are most frequent, most of them are stage I and need no further therapy than nephrectomy.
  - Mesoblastic nephroma usually occurs in young babies. It is a benign tumor that needs no further therapy than nephrectomy. In children with a very cellular mesoblastic nephroma and for children more than 12 months of age, a careful follow up is necessary.
2. High-risk cases are rare. If necessary, the reduction of tumor size with Vincristine alone given before nephrectomy is useful in making the nephrectomy less dangerous and for avoiding high-stage tumors.
3. Babies with large Stage II or III tumors present the same problems as those found in tumors of this type in older children. Theoretically, chemotherapy should be the same as for older children but actinomycin D can cause severe side-effects in the newborn or young babies and doses should be reduced to two-third. For Stage II, post-operative treatment according to Stage I is recommended for 18 weeks.
4. In case of Stage II + and III, AVE protocol with dose reduction is recommended, but abdominal irradiation is poorly tolerated by infants and sequelae are severe.

## DISCUSSION

Great strides have been made in the treatment of Wilms' tumor although high-grade Wilms' tumor still needs further studies and improvement in outcomes. Continued follow up of “cured” patients will allow further identification and quantization of late complications of treatment. The high success rate is an accomplishment in treatment of Wilms' tumor, and it serves as an excellent model and encouragement for treatment of other childhood cancers. Perhaps the most fundamental question in the management of a suspected malignant renal tumor in a child is the timing of surgery, which this review article intends to address.

NWTS recommends upfront surgery – nephrectomy. A trans-abdominal, trans-peritoneal incision is recommended to permit the inspection of sites of involvement and to facilitate the biopsy of suspicious sites.[6] Pre-operative chemotherapy as per NWTS 5 studies is indicated when Wilms' tumor occurs in a solitary kidney, when it occurs in horseshoe kidney, when there is tumor thrombus in the inferior vena cava above the level of the hepatic veins, and when the child presents with respiratory distress due to extensive pulmonary metastases.[31]

The SIOP recommends upfront chemotherapy to all patients, including the stage 1 Wilms' tumor, except for children with less than 6 months of age.

Both these approaches have their own merits and demerits, and it is imperative to understand these before adopting a protocol as a regime for management of children with Wilms' tumor.

Histologic classification is a major issue for debate since the time these study groups have started. In NWTS, upfront nephrectomy provides for the entire kidney for histological evaluation and an accurate risk stratification based on presence or absence of anaplasia, whereas in SIOP, patients do not even undergo tumor biopsy/FNAC before starting therapy. On SIOP 93-01, approximately 5% of lesions in patients treated with chemotherapy were ultimately shown not to be Wilms' tumor and included 1.8% that were benign.[12] Other studies estimating the risk of giving chemotherapy to patients without a pre-operative biopsy and thus patients with benign disease receiving chemotherapy showed this risk to be up to 7.6% to 9.9%[32] reducing to the recent Figures in the SIOP-6 and SIOP-9 trials, respectively, affecting only 1.5% and 1.6% of the referred patients.[29,33] The only histology available in SIOP study group is post-nephrectomy, which is performed 4 weeks after chemotherapy. Here, the debatable issue has always been whether pre-operative chemotherapy alters the entire histology of Wilms' tumor. To a certain extent, the histology is altered with chemotherapy with the necrosis of the tumor cells; however, the anaplastic features of the tumor are notable here, which are important for risk stratification and do not regress with pre-operative chemotherapy. Up front nephrectomy has a research benefit in allowing the untreated tumor to be studied in terms of the tumor's molecular biology, whereas the benefit of SIOP approach lies in reduction of tumor volume and down-staging the tumor and thus reducing the chances of intra-operative tumor spillage.[34] As a result, fewer patients received local irradiation on SIOP-9 than on NWTS-5 although slightly more of the SIOP-9 patients received anthracycline.[18] A second advantage of preoperative chemotherapy is that response to treatment may provide a valuable prognostic indicator.[35,36] Chemotherapy plays an undisputed role in management of Wilms' tumor. The activity of dactinomycin and vincristine against Wilms' tumor was shown in the 1950s and 1960s, and these drugs have served as the cornerstone of Wilms' tumor therapy ever since.[37,38] Doxorubicin was added to the Wilms' tumor treatment protocol in the 1970s.[39] With the conclusions of the trials conducted by SIOP and NWTS, it is clear that the duration of chemotherapy has been reduced drastically, e.g., 6 months of post-operative chemotherapy instead of 15 months for Stage II to IV is sufficient as per NWTS 4 study,[30] and 4 instead of 8 weeks for Stage I tumors as per SIOP 93-01 study.[33] Progressive research and trails have optimized the use of chemotherapeutic agents and in future too, there will be many more modifications in the use of these agents so that acute as well as long-term toxicities are lessened to improve the quality of life of the children affected by Wilms' tumor.

Very few detailed studies are available from India, and the ones available follow the NWTS protocol.[39,40] Few centers have tried with upfront chemotherapy in Wilm's tumor patients with FNAC confirmation of the diagnosis as per the UKCCSG protocol.[41] To date, no published reports have been found regarding the outcome of WT as per the SIOP protocol in our country.

## SUMMARY

The success in the treatment of Wilms' tumor over decades is a tremendous achievement in oncology. Modern treatment regimens yield overall survival (OS) rates of 90%, and this success has precipitated a shift in emphasis to reducing toxicity. Although North America (NWTS) and Europe (SIOP, UKCCSG) have different philosophies on preoperative chemotherapy, the overriding message is that most patients with Wilms' tumor survive long term, regardless of the sequence of therapeutic interventions. There still remains a “problematic” group, which includes anaplastic, bilateral or recurrent disease, and this group does not show the same response to treatment with the current protocols and regimes. This “problematic” group accounts for about 25% of patients in the Western Literature, (definitely would be much higher in our country) and this high proportion highlights the need for a continued effort to develop novel treatment approaches. For developing countries such as India, there is no data available as to the similarities or dissimilarities in the biologic and therapeutic behavior of Wilms' tumor as compared with the Western world. A detailed and extensive study in this area remains a great future potential. Moreover, in our country, the incidence of advanced and metastatic Wilms' tumor is considerably higher than in the western literature as the patients usually present in the late stages with large tumors and many a times with metastates. The other problem in a developing nation such as ours is the lack of resources and facilities for histopathological diagnosis on FNAC/biopsy. Considerable amount of time and resources are required if the child has to undergo all the investigations to procure a diagnosis of WT, which complicates matters further with the already delayed presentation of the child with WT. The pretreatment investigation protocol suggested by SIOP mandates clinical examination, an ultrasonographic evaluation of the tumor characteristics and an intravenous pyelogram for beginning therapy, although in the advanced era computed tomography and MR imaging with pyelography can be performed. In our Indian circumstances where more than these basic investigations may be difficult to perform, probably it is more appropriate and feasible to follow the SIOP protocol for management of Wilms' tumor with the added advantage of preventing intra-operative tumor spillage with upfront chemotherapy.

## FUTURE DIRECTIONS IN INDIA

The Surgical Oncology Section of the Indian Association of Pediatric Surgeons intends to study this tumor on a nationwide basis with regard to various aspects and analyze the differences in the presentation and management characteristics of Wilms' tumor as compared with the Western Literature. The Indian Wilms' Tumor Study Group (IWTSG) will allow us to evaluate our own standards and set new parameters for the future.
